# Combing MRI Perfusion and ^18^F-FDG PET/CT Metabolic Biomarkers Helps Predict Survival in Advanced Nasopharyngeal Carcinoma: A Prospective Multimodal Imaging Study

**DOI:** 10.3390/cancers13071550

**Published:** 2021-03-28

**Authors:** Sheng-Chieh Chan, Chih-Hua Yeh, Joseph Tung-Chieh Chang, Kai-Ping Chang, Jen-Hung Wang, Shu-Hang Ng

**Affiliations:** 1 Department of Nuclear Medicine, Hualien Tzu Chi Hospital, Buddhist Tzu Chi Medical Foundation, Hualien 970473, Taiwan; wi110911@tzuchi.com.tw; 2 Department of Medicine, School of Medicine, Tzu Chi University, Hualien 97004, Taiwan; 3 Department of Diagnostic Radiology, Linkou Chang Gung Memorial Hospital and Chang Gung University, Taoyuan 333423, Taiwan; foliatus@cgmh.org.tw; 4 Department of Radiation Oncology, Linkou Chang Gung Memorial Hospital and Chang Gung University, Taoyuan 333423, Taiwan; jtchang@cgmh.org.tw; 5 Department of Otorhinolaryngology, Linkou Chang Gung Memorial Hospital and Chang Gung University, Taoyuan 333423, Taiwan; changkp@cgmh.org.tw; 6 Department of Medical Research, Hualien Tzu Chi Hospital, Buddhist Tzu Chi Medical Foundation, Hualien 970473, Taiwan; paulwang@tzuchi.com.tw

**Keywords:** multiparametric magnetic resonance imaging, diffusion magnetic resonance imaging, PET/CT, head and neck neoplasms, prognosis

## Abstract

**Simple Summary:**

Among various types of head and neck cancers, nasopharyngeal carcinoma (NPC) has a distinct epidemiology and prognosis. Tumor recurrence in NPC poses significant clinical challenges and identifying novel prognostic biomarkers is crucial. Imaging biomarkers derived from positron emission tomography (PET) and magnetic resonance imaging (MRI) reflect different biological facets of tumors. However, whether pretreatment PET and MRI imaging biomarkers can complement each other in the survival prediction of NPC patients has not been determined. This prospective study aimed to analyze the prognostic value of imaging biomarkers derived from pretreatment MRI and PET in NPC. The model integrating the MRI perfusion markers with PET metabolic parameter demonstrated higher prognostic performance than the traditional staging system and individual model with either MRI or PET alone. A staging work-up for NPC including both functional MRI and PET parameters may provide clinicians with supplementary prognostic information in addition to morphologic assessment of tumor extent.

**Abstract:**

We prospectively investigated the prognostic value of imaging parameters for nasopharyngeal carcinoma (NPC) using dynamic contrast-enhanced MRI (DCE-MRI), diffusion-weighted imaging (DWI), and 2-deoxy-2-[fluorine-18]fluoro-D-glucose positron emission tomography (^18^F-FDG PET)/computed tomography (CT). Patients with stage III–IVb NPC underwent F-FDG PET/CT, DCE-MRI, and DWI before treatment. Kaplan-Meier and Cox-regression analyses were used to assess associations of PET and MRI imaging biomarkers with overall survival (OS) and recurrence-free survival (RFS). We used independent prognosticators to establish prognostic models; model performance was examined using Harrell’s concordance index (c-index). Sixty-one patients were available for analysis, as 13 patients died and 20 experienced recurrence. Total lesion glycolysis (TLG) (*p* = 0.002) from PET/CT and the initial area under the curve (iAUC) (*p* = 0.036) from DCE-MRI were identified as independent prognosticators of OS; Epstein-Barr virus (EBV) DNA (*p* = 0.027), the extracellular volume fraction (*V_e_*) (*p* = 0.027) from DCE-MRI, and TLG/iAUC (*p* = 0.025) were significant predictors of RFS. The c-indices of the prognostic models incorporating TLG + iAUC in predicting OS and incorporating EBV DNA + *V_e_* + TLG/iAUC in predicting RFS were 0.79 and 0.76, respectively. These were significantly higher than the corresponding c-indices of the TNM staging system (*p* = 0.047 and 0.025, respectively); they were also higher than those of models with only MRI or PET biomarkers. In conclusion, the combination of pretreatment DCE-MRI and ^18^F-FDG PET/CT imaging biomarkers helps survival prediction in advanced NPC. Integrating MRI perfusion with PET metabolism and plasma EBV information may aid clinicians in planning the optimal personalized management strategy.

## 1. Introduction

Among various types of head and neck cancers, nasopharyngeal carcinoma (NPC) is a malignancy with a distinct epidemiology and prognosis [1,2]. Although NPC is sensitive to chemoradiotherapy [3], recurrence occurs in approximately 30% of patients presenting with advanced-stage disease and continues to pose significant clinical challenges [2,4,5]. Thus, identifying novel prognostic biomarkers is crucial to improve the management of these patients.

Recently, in cancer research, there has been increasing interest in imaging biomarkers detected by magnetic resonance imaging (MRI) and positron emission tomography (PET) [6,7,8]. The cellularity or perfusion of the tumor can be noninvasively assessed using diffusion-weighted imaging (DWI) and dynamic contrast-enhanced MRI (DCE-MRI), respectively. The apparent diffusion coefficient (ADC) calculated via DWI has been shown to be related to disease-free survival in NPC [9]. A DCE-MRI-based study has demonstrated that early changes in the kinetic parameters could predict the response to chemotherapy in NPC patients [10]. To date, 2-deoxy-2-[fluorine-18]fluoro-D-glucose (^18^F-FDG) PET/CT parameters, including the standardized uptake value (SUV) and total lesion glycolysis (TLG), which provide functional information on tumor glucose metabolism, have shown promise in predicting the treatment response and survival of NPC patients [11].

Imaging biomarkers derived from ^18^F-FDG PET/CT, DWI, or DCE-MRI reflect different biological facets of malignant tumors. Therefore, these imaging biomarkers may play complementary roles in the management of malignancy. However, there is still insufficient data regarding the prognostic implications of combining the imaging biomarkers provided by both PET/CT and MRI in NPC.

This prospective study aimed to analyze the prognostic value of imaging biomarkers derived from pretreatment PET/CT, DCE-MRI, and DWI in advanced NPC. We hypothesized that a combination of imaging biomarkers on PET/CT and MRI might help predict the prognosis of locoregionally advanced NPC patients.

## 2. Materials and Methods

### 2.1. Study Patients

Patients with histologically confirmed NPC were deemed eligible. The exclusion criteria were: (1) pregnant or lactating women, (2) patients with synchronous malignancy or recurrent tumors, and (3) patients with contraindications to MRI. Ethical approval was granted by the local Institutional Review Board (IRB no.100-4365C) and the study was conducted in accordance with the Declaration of Helsinki. Between August 2011 and July 2014, we identified 81 potentially eligible patients with stage III–IVb cancer. The study participants underwent both ^18^F-FDG PET/CT and head-neck MRI with DCE-MRI and DWI before chemoradiotherapy. All examinations were performed within 14 days. Twenty patients were excluded from the final analysis: 16 had primary tumors with soft tissue components that were too thin or small for achieving optimal DCE-MRI or DWI results, and the other four were lost to follow-up. Consequently, the data of 61 patients were available for analysis (Table 1). Some overlap was found between our patient group and the cohort of our previous study which evaluated the predictive role of interim PET/CT during chemoradiation [12].

### 2.2. Multimodal MRI

We performed the MRI examinations using a 3-T scanner (Magnetom Trio with TIM, Siemens, Erlangen, Germany). Conventional MRI of the head and neck region was performed in the axial and coronal projections using the following sequences: T2-weighted turbo spine echo (TSE) with fat saturation, T1-weighted TSE, and postcontrast fat-saturated T1-weighted TSE. DWI was performed using single-shot spin-echo echo-planar imaging with a modified Stejskal-Tanner diffusion gradient pulsing scheme. We applied motion-probing gradients (b-value = 800 s/mm^2^) along the three orthogonal directions. The repetition time (TR) and echo time (TE) were 8200 ms and 84 ms, respectively. We performed DCE- MRI using a 3D T1-weighted spoiled gradient-echo sequence with the following parameters: TR/TE = 3.5/1.13 ms, a 230 × 230-mm field of view, and a 108 × 128 matrix. A spatial saturation slab was implanted below the targeted region to minimize the inflow effect from the carotid arteries. Before the administration of the contrast agent, baseline longitudinal relaxation time (T10) values were calculated from images acquired with different flip angles (4°, 8°, 15°, and 25°). The dynamic series involved the use of the same sequence with a 15° flip angle. After four acquisitions using dynamic baseline scanning, a standard dose (0.1 mmol/kg body weight) of gadopentetate dimeglumine was administered using a power injector through a cannula placed in the antecubital vein (rate = 3 mL/s), which was immediately followed by a saline flush. A total of 80 volumes were acquired (temporal resolution = 3.3 s).

### 2.3. ^18^F-FDG PET/CT

Before the ^18^F-FDG PET/CT imaging, all the study participants had to fast for at least 6 h. The scans were performed using a Biograph mCT system (Siemens Medical Solutions, Malvern, PA, USA). The system consisted of a four-ring PET scanner with a transaxial field of view of 70 cm, an axial field of view of 22.1 cm, and a 40-section CT scanner. Subsequently, 50–70 min following the injection of ^18^F-FDG (370 MBq), PET scans were performed from the vertex to the mid-thigh. The scanning time per table position was 1.5 min and the imaging matrix size was 200 × 200. All the patients had a plasma glucose level of <150 mg/dL before the ^18^F-FDG injection. Before the PET scan, a standard helical CT scan was performed from the head to the proximal thighs using the manufacturer’s dose reduction software. The preset was 120 kV with 40 × 0.6 mm collimation, a pitch of 1.5, and a 2 mm slice thickness. No intravenous iodinated contrast agent was administered. We reconstructed the PET images using an ordered subsets expectation maximization iterative reconstruction algorithm (two iterations, 21 subsets) and the CT data for attenuation correction.

### 2.4. PET Image Analysis

We performed tumor segmentation on PET images using the PMOD 3.2 software package (PMOD Technologies Ltd., Zurich, Switzerland). First, we drew tumor boundaries of nasopharyngeal tumor (blinded to clinical data) with the aid of the T2-weighted MR images and the T1-weighted post-contrast MR images to avoid the inclusion of the physiological FDG uptake. Second, the tumor boundaries were determined using a fixed SUV threshold of 2.5. The voxels associated with an SUV larger than this threshold within the contouring boundary were incorporated into the tumor volume. Finally, the SUV and TLG were calculated by the software.

### 2.5. MR Image Analysis

The ADC values were measured on ADC maps by drawing the regions of interest (ROI) on the nasopharyngeal tumors. The DCE-MRI images were transferred to a post-processing workstation and were analyzed using a dedicated software application (TISSUE 4D, Siemens Medical Systems, Erlangen, Germany). After the motion correction and registration of the pre- and post-contrast acquisitions, T1 mapping was generated automatically. The ROIs were manually drawn onto the primary nasopharyngeal tumor images. The software allows for the implementation of a population-based approach for the determination of the arterial input function (AIF) that was scaled based on the gadolinium dose and modeled using the bi-exponential method proposed by Tofts and Kermode [13]. The following pharmacokinetic parameters were subsequently calculated: the volume transfer constant (*K^trans^*), rate constant (*K_ep_*), extravascular extracellular volume fraction (*V_e_*), and initial area under the curve (iAUC).

### 2.6. Treatment and Follow-Up

The patients underwent external beam radiotherapy with a cumulative dose of at least 64.8 Gy. Concurrent cisplatin-based chemotherapy involving intravenous cisplatin (50 mg/m^2^ on day 1) and oral tegafur (800 mg/day) plus oral leucovorin (60 mg/day from day 1 to day 14) was also administered to the patients. The chemotherapy schedule was repeated every 2 weeks throughout the course of radiation therapy [14]. Patients underwent clinical follow-up examinations every week during the treatment, every 3 months in the first 2 years after the treatment, every 4 months in the subsequent 2 years, and every 6 months thereafter. A follow-up MRI was performed 3 months after the completion of the treatment. Thereafter, an MRI or CT scan was performed every 6 months or in the presence of clinical deterioration.

### 2.7. Outcome Determination and Statistical Analysis

The patients were followed-up with until July 2019 or death. Overall survival (OS) and recurrence-free survival (RFS) served as the main outcome measures. The OS period was defined as the duration from the date of diagnosis until the date of death or was censored at the date of the last follow-up in surviving patients. The RFS period was calculated as the time between the end of chemoradiotherapy and the tumor recurrence date or was censored at the date of the last follow-up. Serial cut-off points were tested for each imaging biomarker, and the value exhibiting the lowest *p* was chosen as the cut-off in the subsequent analyses. The Kaplan-Meier method with a log-rank test was used for the survival analysis. The effect of each variable on the study’s outcome was first tested via univariate analysis. Subsequently, a Cox regression analysis was performed to identify the independent predictors of survival. Two-tailed *p* values of <0.05 were considered statistically significant. All data were analyzed using SPSS software version 17.0 (SPSS Inc., Chicago, IL, USA).

### 2.8. Performance and Validation of the Prognostic Model

Further, we constructed prognostic models for OS and RFS based on independent risk factors. The prognostic performances of the models and TNM staging system were examined by Harrell’s concordance index (c-index) [15]. We performed a validity assessment of the prognostic models using the bootstrap method for internal validation. The validation process was performed with 1000 bootstrap samples consisting of random samples obtained from the original sample with replacement. The results of the bootstrapping validation were expressed as bias with the 95% confidence interval and significance (*p*-value). The analysis was performed using R version 3.4.2.

## 3. Results

The median follow-up period was 3.79 years (range, 0.14–5.6 years) for the entire cohort and 4 years (range, 2.81–5.6 years) for the surviving patients. At the time of the last follow-up, 13 (21.3%) deaths had occurred. Disease recurrence was observed in 20 (32.8%) patients. The OS and RFS rates were 83.6% and 79.4% at 3 years, respectively, and were 78.4% and 48.0% at 5 years, respectively.

### 3.1. PET/CT and MRI Imaging Biomarkers for Predicting Survival Endpoints

The results of the univariate analysis of the clinical variables and ^18^F-FDG PET, along with survival-related MRI imaging biomarkers, are summarized in Table 2. According to the univariate analysis, the tumor (T) classification, TLG, *K^trans^*, *K_ep_*, iAUC, and TLG/iAUC were identified as significant predictors for OS. Because iAUC had a high degree of collinearity with TLG/iAUC, each one of these parameters was entered separately into the multivariate analysis model (with TLG and iAUC versus TLG/iAUC). TLG (*p* = 0.002, hazard ratio (HR) = 6.7) with iAUC (*p* = 0.036, HR = 0.3) or TLG/iAUC (*p* = 0.002, HR = 6.13) were identified as independent predictors of OS based on each analysis model, respectively, following the adjustment for potential confounders in the multivariate analysis (Table 3).

**Figure 1:**
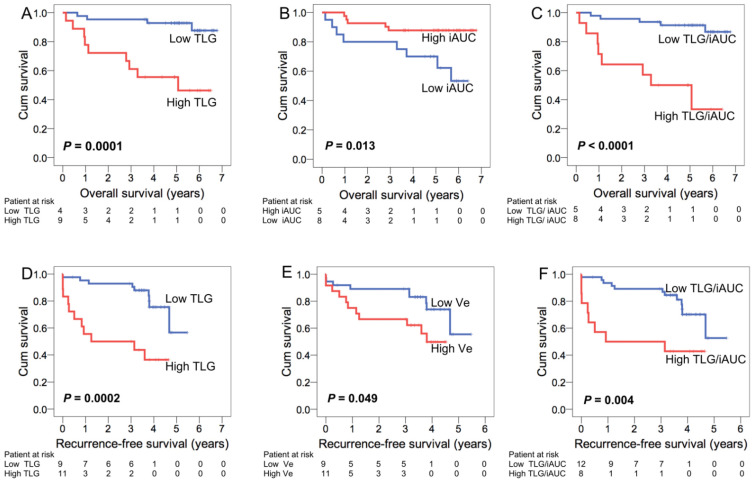
Kaplan-Meier plots of advanced nasopharyngeal carcinoma (NPC) patients stratified based on high and low levels of the independent imaging biomarkers. TLG (**A**), iAUC (**B**), and TLG/iAUC (**C**) were identified as significant predictors of overall survival. TLG (**D**), *Ve* (**E**), and TLG/iAUC (**F**) were as significant factors for the analysis of recurrence-free survival.

In the univariate analysis of RFS, the T classification, EBV DNA load, TLG, ADC_min_, *V_e_*, and TLG/iAUC were identified as significant factors. The EBV DNA load (*p* = 0.049, HR = 2.65) and TLG (*p* = 0.002, HR = 4.41) retained independent prognostic significance for RFS in the Cox-regression model with TLG and iAUC, while the EBV DNA load (*p* = 0.027, HR = 3.05), *V_e_* (*p* = 0.027, HR = 2.85) and TLG/iAUC (*p* = 0.025, HR = 2.92) retained their independent significance for RFS in the Cox-regression analysis model with TLG/iAUC. Figure 1 compares the Kaplan-Meier plots representing patient survival based on different independent imaging biomarkers.

### 3.2. Performance and Validation of the Prognostic Models

**Figure 2:**
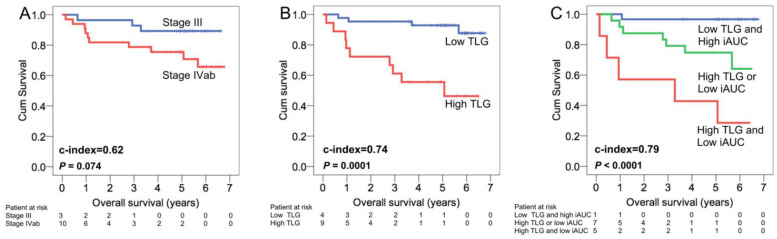
Comparison of patient overall survival stratified based on the TNM stage (**A**), PET biomarker, TLG (**B**), and the prognostic model incorporating both PET and MRI biomarkers, the TLG and iAUC (**C**). This combined model enabled a better survival stratification with a higher c-index compared with that of TLG alone. It also exhibited a significantly higher c-index than that of the TNM system. TLG, total lesion glycolysis; iAUC, initial area under the curve.

We further formulated prognostic models by summing up the independent prognosticators identified in the multivariate analysis for each prognostic endpoint. Each prognostic factor was assigned a value of 0 when it was found to be absent or 1 when it was present. Table 4 presents Harrell’s c-indices of the prognostic models in comparison with those of the TNM stage and singular imaging biomarker assessment. The model combining TLG with iAUC demonstrated a c-index of 0.79 in predicting OS, which was significantly higher than the c-index of the TNM staging system (0.62, *p* = 0.047; Figure 2) and higher than the c-index of the TLG (0.74) or iAUC (0.65) assessment. For RFS, the model integrating EBV DNA, *V_e_*, and TLG/iAUC had a c-index of 0.76. The c-index of this model was higher than that of the TNM staging system (0.61, *p* = 0.025), the TLG (0.71) or iAUC (0.52) assessment alone, or the model integrating EBV DNA with TLG (0.74)

Lastly, in the bootstrapping validation, the c-index of the model based on TLG with iAUC or EBV DNA, *V_e_*, and TLG/iAUC was comparable to those in the training cohort (Table 5).

## 4. Discussion

For the staging of primary NPC, MRI is the imaging method of choice whereas ^18^F-FDG PET/CT is mainly used to detect metastasis. According to previous reports [9,10,11], each modality has shown its predictive value in prognosis separately. However, the issue whether a combination of pretreatment PET/CT and MRI imaging biomarkers can aid in the survival prediction of NPC patients has not been determined. In this study, we noted that the TLG and iAUC had high prognostic power in predicting OS while the EBV DNA, *V_e_*, and TLG/iAUC independently predicted RFS. The prognostic models incorporating both PET and MRI biomarkers demonstrated higher c-indices in predicting OS and RFS than models with the TNM system. Our results support our hypothesis that a combination of PET metabolism and MRI perfusion biomarkers help to provide better prognostic prediction in advanced NPC patients.

DCE-MRI is an imaging technique for the pharmacokinetic analysis of a specific tumor portion. In the univariate analysis, we found that the iAUC, *K^trans^*, and *K_ep_* were significantly associated with OS, whereas the *V_e_* predicted RFS. iAUC is related to both tumor perfusion and permeability [16]. A low iAUC value reflects decreased blood volume within the tissue, hindering the delivery of chemotherapeutic agents to the tumor. In a study of unresectable gastric cancer by Lee et al., the iAUC value of the tumors was significantly lower in non-responders to chemotherapy [17]. Huang et al. analyzed the usefulness of DCE-MRI in predicting the prognosis of non-small cell lung cancer treated by chemoradiotherapy and found that non-responders had a lower baseline iAUC [18]. Our data further demonstrated a positive association of pretreatment iAUC and OS in NPC. On the other hand, the *V_e_* reflects the extravascular extracellular space. The extracellular space in a malignant tumor is characterized by a larger interstitial space, higher collagen concentration, and higher interstitial fluid pressure [19]. Koh et al. reported that the *V_e_* is an independent predictor of OS in primary colorectal cancer [20]. In a study by Chin et al., a positive association was found between the *V_e_* and incidence of early distant metastases in NPC [21]. Our findings further support that the *V_e_* negatively prognosticated survival in NPC.

*K^trans^* is associated with the vascularity and permeability of the tumor and tumoral vessel whereas *K_ep_* represents the backflow rate constant from the tissue into the vessel, relating to tissue vascular permeability and surface area. In an assessment of head and neck squamous cell carcinoma, Chawla et al. reported that patients with a lower *K^trans^* had a shorter period disease-free survival [22]. In a recent study, it was found that a tumor with a higher *K_ep_* independently prognosticated OS in patients with oropharyngeal or hypopharyngeal cancer [23]. Although both *K^trans^* and *K_ep_* in this study were significantly associated with OS in the univariate analysis, they did not retain their independent prognostic significance in the multivariate analysis as iAUC did. Consequently, iAUC appeared to be a better DCE-MRI predictor of OS than *K^trans^* and *K_ep_* in locoregionally advanced NPC patients.

DWI facilitates the evaluation of the diffusivity of water molecules in the tumour tissues by quantifying via ADC, which is inversely correlated with cell density. Yan et al. investigated the prognostic value of the pretreatment ADC in NPC [24] and reported that patients with a high pretreatment ADC exhibited longer OS and locoregional failure-free survival. In this study, we also noted that a higher ADC was associated with a better RFS in univariate analysis, but lost its independent prognostic significance in multivariate analysis. In another report, however, a higher pretreatment ADC prognosticated a poorer disease-free survival rate in NPC patients [9]. Further studies are warranted in order to clarify these inconsistencies.

Previous studies have demonstrated that the TLG is an independent predictor for disease recurrence or survival in NPC [11,12,25,26,27]. A meta-analysis by Li et al. further confirmed that a higher TLG reflects a negative prognostic value and that the TLG is significantly associated with both OS and RFS in NPC patients [26]. Our results reinforced the idea that a high TLG is a strong prognosticator for both OS and RFS in locoregional advanced NPC, with hazard ratios of 6.696 and 4.414, respectively.

In this report, we also noted that the TLG/iAUC ratio was a strong prognostic predictor. Advanced NPC is usually characterized by a bulky tumor with high glycolysis and altered vasculature. The increased proliferation of cancer cells may result from the promotion or maintenance of the cancer vascular system via up-regulated radiation-induced vascular endothelial growth factor production [28]. Recently, a phase II multicenter trial demonstrated that the administration of bevacizumab with chemoradiotherapy may delay the progression of subclinical distant cancer in patients with locoregionally advanced NPC [29]. TLG/iAUC appeared to be a biomarker integrating metabolism and perfusion information of a tumor and could be considered as an important predictor in future trials that investigate angiogenetic agents in NPC.

**Figure 3:**
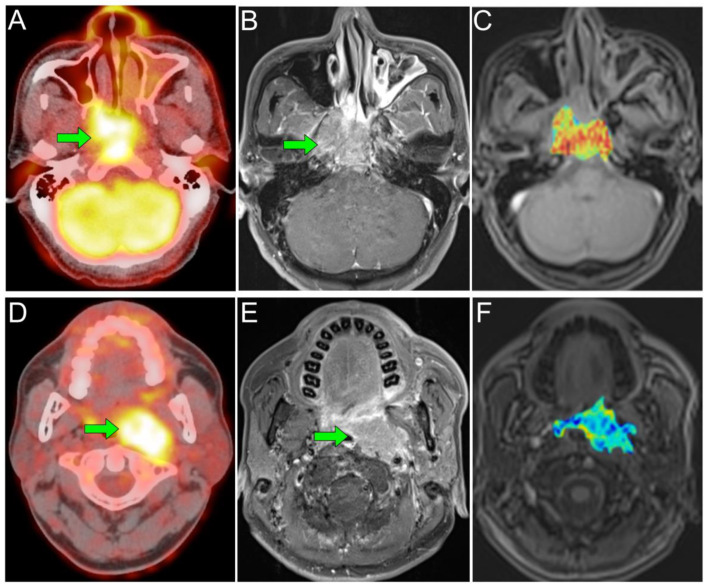
Cases with different combinations of TLG and iAUC. (**A**–**C**) Representative axial PET or MRI images of a primary NPC patient with T4N1 disease. (**A**) ^18^F-FDG PET/CT image; (**B**) contrast-enhanced T1-weighted MR image; and (**C**) an iAUC color map. This patient had a low TLG of 130.14 g/mL × mL and a high iAUC of 734.94. He survived without disease recurrence for 6.04 years after diagnosis. (**D**–**F**) Axial PET and MRI images of a primary NPC patient with T3N1 disease. This tumor extended downward to the oropharynx. (**D**) ^18^F-FDG PET/CT image; (**E**) contrast-enhanced T1-weighted MR image; and (**F**) an iAUC color map. He had a high TLG of 265.41 g/mL × mL and a low iAUC of 522.50. Despite concurrent chemoradiotherapy, this patient died of distant recurrence with a short overall survival period of 3.28 years. iAUC, initial area under the curve; TLG, total lesion glycolysis.

To determine the prognostic implication of these imaging biomarkers, we further formulated prognostic models by combining the independent prognostic factors. The model combining the TLG with iAUC exhibited a significantly higher c-index than the TNM stage classification in predicting OS (Figure 2 and Figure 3). Additionally, the model integrating the EBV DNA, *V_e_*, and TLG/iAUC exhibited a superior c-index to that of the TNM system in RFS prediction (Table 4). Tumor vascularity, glucose metabolism, and EBV DNA load may have a synergistic effect on the biological aggressiveness or metastasis of NPC in endemic areas. The latent infection of NPC cells by EBV and expression of latent EBV genes have been reported to drive glucose metabolism and to modify the tumor microenvironment, thereby enhancing the malignant properties including immune evasion and anti-apoptosis [30]. Therefore, a model comprised of three biomarkers of perfusion, metabolism, and EBV DNA load could help to develop a deep understanding of the prognosis of NPC patients. In clinical practice, our proposed model may help develop a comprehensive tool for identifying a subgroup of NPC patients at high risk of poor survival. Consequently, these patients should be closely followed-up with after treatment to achieve early detection of potentially salvageable lesions. These subjects may also be considered as potentially suitable candidates for future trials of novel treatment approaches including anti-angiogenetic therapy.

In our previous studies on the predictive role of pretreatment perfusion MRI in NPC patients, it was noted that Ve was associated with the distant metastasis rate [20] and treatment failure [31]. In another study on NPC that assessed the role of interim PET/CT during chemoradiation [12], we found that a combination of interim PET/CT and EBV titer helped predict the treatment outcome. In the current study, we further investigated the utility of pretreatment PET and MRI in predicting long-term survival in advanced NPC patients and found that combining perfusion MRI parameters (Ve and iAUC) with the TLG value on PET had a higher c-index than that of TNM staging in the prognostic assessment.

The EBV load has been recognized as a promising prognostic biomarker for the NPC patients. EBV adopts a specific form of latency infection in NPC cells, which may promote tumorigenicity by enhancing its invading activity. Pretreatment EBV DNA level are correlated with patient survival and tumor stage [30]. Besides posttreatment EBV DNA titers can reflect the mass of residual cancer and predict recurrence [32].

Of note, the association of EBV infection and NPC varies among countries. The pathogenesis of WHO types II and III NPC is closely linked to the EBV. However, the association of WHO type I tumor and the EBV is weak [30]. WHO type II and III tumors are the predominant types of NPC in endemic areas, whereas many patients in non-endemic regions have WHO type I tumor. This study evaluated NPC patients from Taiwan, therefore its results may be applicable in other endemic regions, but not in nonendemic regions. Nevertheless, EBV load has been reported as sensitive and highly specific in detecting disease recurrence and metastases in NPC patients of Western country [33], while EBV-related nonkeratinizing differentiated NPC is increasing across all sexes and races in the United States [34]. Our study may still be informative for those WHO Type II–III NPC patients in nonendemic regions.

Other limitations of our study merit consideration. First, this was a single-center study, and the sample population might not be large enough for global generalization. Second, a fixed SUV was used for tumor contouring which may not consistently map the tumor’s actual extent; however, this approach is in line with the one used in most studies investigating the prognostic utility of PET parameters in NPC, thereby enabling a practical comparison with results presented in the literature [11,25]. A set SUV of 2.5 for the volume of interest delineation was adopted in eight of 10 studies in one me-ta-analysis [26]. Further investigations with a multicenter design and external validation are necessary to confirm our results.

## 5. Conclusions

In summary, this study showed that the model integrating the PET metabolo-volumetric parameter with the MRI perfusion markers demonstrated higher prognostic performance than the traditional staging system. Combining dynamic contrast-enhanced MRI and ^18^F-FDG PET/CT imaging biomarkers helps to predict survival in advanced NPC, and would facilitate a comprehensive clinical management strategy. Thus, a staging work-up for advanced NPC with both PET/CT and MRI may provide clinicians with more complete prognostic information in addition to information regarding tumor extent.

## Figures and Tables

**Table 1:** General characteristics of the study participants (*n* = 61).

Variable	Number of Patients (%)
Age (years), median ± SD	46 ± 13
Sex	
Male	50 (82)
Female	11 (18)
Histology, n (%)	
Non-keratinizing carcinoma	3 (5)
Undifferentiated carcinoma	58 (95)
Overall stage	
III	28 (46)
IVa	21 (34)
IVb	12 (20)
T classification	
T1	13 (21)
T2	2 (3)
T3	19 (31)
T4	27 (45)
N classification	
N0	3 (5)
N1	20 (33)
N2	26 (42)
N3	12 (20)
EBV copy number (mL^−1^)	
Mean ± SD	18,486 ± 65,394

Data are expressed as counts and percentages (in parentheses), unless otherwise indicated. SD = standard deviation.

**Table 2:** Univariate analysis of clinical factors and PET or MRI imaging biomarkers in relation to overall survival and recurrence-free survival in primary advanced NPC patients.

Parameter	Patient No.	OS		RFS	
		5-Year OS (Event No.)	*p* Value	5-Year RFS (Event No.)	*p* Value
Age (years)			0.073		0.641
≤46	32	82.4 (4)		41.8 (10)	
>46	29	67.1 (9)		63.4 (10)	
Sex			0.793		0.107
Male	50	78.6 (11)		39.1 (19)	
Female	11	81.8 (2)		90.9 (1)	
Tumor stage			0.074		0.247
III	28	89.3 (3)		33.7 (7)	
IVa-b	33	70.8 (10)		59.0 (13)	
T classification			0.007		0.017
T1–3	34	93.3 (1)		49.6 (7)	
T4	27	74.6 (12)		49.9 (13)	
N classification			0.581		0.685
N 0–1	23	82.1 (4)		31.2 (9)	
N 2–3	38	77.9 (9)		65.5 (11)	
Histology			0.408		0.890
Non-keratinizing carcinoma	3	100 (0)		66.7 (1)	
Undifferentiated carcinoma	58	74.7 (13)		47.7 (19)	
EBV DNA load (copies/mL)			0.331		0.022
≤2750	31	83.6 (5)		55.5 (6)	
>2750	30	75.3 (8)		44.7 (14)	
Imaging biomarker					
TLG (g/mL × mL)			0.0001		0.0002
≤170	43	87.7 (4)		56.6 (6)	
>170	18	46.3 (9)		36.5 (14)	
SUV_max_ (g/mL)			0.419		0.505
≤11	32	71.2 (8)		70.1 (9)	
>11	29	81.3 (5)		27.0 (11)	
ADC_min_ (10^−6^ mm^2^/s)			0.191		0.01
≤200	16	62.5 (5)		38.5 (9)	
>200	45	79.9 (8)		48.9 (11)	
*K^trans^* (10^−3^ min^−1^)			0.015		0.916
≤680	16	60.2 (7)		64.8 (5)	
>680	45	86.7 (6)		47.8 (15)	
*K_ep_* (10^−3^ min^−1^)			0.041		0.081
≤950	37	75.4 (11)		0 (15)	
>950	24	91.7 (2)		76.0 (5)	
*V_e_* (10^−3^)			0.224		0.049
≤140	37	80.7 (6)		55.4 (9)	
>140	24	68.8 (7)		49.8 (11)	
iAUC			0.013		0.952
≤650	20	53.3 (8)		66.1 (6)	
>650	41	87.8 (5)		47.3 (14)	
TLG/iAUC			<0.0001		0.004
≤0.26	47	86.8 (5)		52.6 (12)	
>0.26	14	33.3 (8)		42.9 (8)	

OS = overall survival; RFS = recurrence-free survival; EBV = Epstein-Barr virus; TLG = total lesion glycolysis; SUV_max_ = maximum standardized uptake value; ADC_min_ = minimum apparent diffusion coefficient; *K*^trans^ = volume transfer constant; *K*_ep_ = flux rate constant; *V*_e_ = extracellular volume ratio; iAUC = initial area under curve; PET = positron emission tomography; MRI = magnetic resonance imaging.

**Table 3:** Multivariate cox-regression analysis of clinical factors and imaging biomarkers relative to overall survival and recurrence-free survival.

Risk Factor	Cox-Regression Analysis with TLG and iAUC				Cox-Regression Analysis with TLG/iAUC			
	OS		RFS		OS		RFS	
	HR (95% CI)	*p* Value	HR (95% CI)	*p* Value	HR (95% CI)	*p* Value	HR (95% CI)	*p* Value
T classification	-	ns	-	ns	-	ns	-	ns
EBV DNA load	-	N/A	2.65 (1.01-6.97)	0.049	-	N/A	3.05 (1.14–8.18)	0.027
TLG	6.70 (2.05–12.87)	0.002	4.41 (1.76–11.09)	0.002	-	N/A	-	N/A
ADC_min_	-	N/A	-	ns	-	N/A	-	ns
*K^trans^*	-	ns	-	N/A	-	ns	-	N/A
*K_ep_*	-	ns	-	N/A	-	ns	-	N/A
*V_e_*	-	N/A	-	ns	-	N/A	2.85 (1.13–7.18)	0.027
iAUC	0.30 (0.10–0.92)	0.036	-	N/A	-	N/A	-	N/A
TLG/iAUC	-	N/A	-	N/A	6.13 (1.91–19.68)	0.002	2.92 (1.14–7.43)	0.025

OS = overall survival; RFS = recurrence-free survival; EBV = Epstein-Barr virus; TLG = total lesion glycolysis; ADC_min_ = minimum apparent diffusion coefficient; *K*^trans^ = volume transfer constant; *K*_ep_ = flux rate constant; *V*_e_ = extracellular volume ratio; iAUC = initial area under curve; HR = hazard ratio; CI = confidence interval; ns = not significant; N/A = not applicable.

**Table 4:** Comparison of Harrell’s concordance index between TNM stage, imaging biomarker assessments, and prognostic models.

Model	OS		RFS	
	c-Index	95% CI	c-Index	95% CI
TNM stage	0.62	0.50–0.75	0.61	0.51–0.72
TLG	0.74	0.63–0.86	0.71	0.60–0.81
iAUC	0.65	0.51–0.78	0.52	0.40–0.63
Prognostic model				
TLG + iAUC	0.79 *	0.68–0.90	N/A	N/A
TLG/iAUC	0.74	0.61–0.86	N/A	N/A
EBV DNA + TLG	N/A	N/A	0.74	0.63–0.84
EBV DNA + *V_e_* + TLG/iAUC	N/A	N/A	0.76 **	0.67–0.85

* *p* = 0.047 in comparison with TNM stage; ** *p* = 0.025 in comparison with TNM stage; CI = confidence interval; N/A = not applicable; OS = overall survival; RFS = recurrence-free survival; iAUC = initial area under curve; TLG = total lesion glycolysis; EBV = Epstein-Barr virus; *V*_e_ = extracellular volume ratio.

**Table 5:** Harrell’s concordance indices of the prognostic models and TNM stage in the training cohort and bootstrapping validation.

Model	Training				Bootstrapping Validation			
	OS		RFS		OS		RFS	
	c-Index	95% CI	c-Index	95% CI	c-Index	95% CI	c-Index	95% CI
TNM stage	0.62	0.50–0.75	0.61	0.51–0.72	0.63	0.51–0.75	0.62	0.46–0.71
Prognostic model								
TLG + iAUC	0.79	0.68–0.90	N/A	N/A	0.80	0.66–0.90	N/A	N/A
EBV DNA + *V_e_* + TLG/iAUC	N/A	N/A	0.76	0.67–0.85	N/A	N/A	0.76	0.66–0.86

CI = confidence interval; N/A = not applicable; OS = overall survival; RFS = recurrence-free survival; TLG = total lesion glycolysis; iAUC = initial area under the curve.

## Data Availability

The data presented in this study are available on request from the corresponding author.

## References

[1] Torre L.A., Bray F., Siegel R.L., Ferlay J., Lortet-Tieulent J., Jemal A. (2015). Global cancer statistics, 2012. CA Cancer J. Clin..

[2] Lee A.W., Ma B.B., Ng W.T., Chan A.T. (2015). Management of Nasopharyngeal Carcinoma: Current Practice and Future Perspective. J. Clin. Oncol..

[3] Lin J.C., Jan J.S., Hsu C.Y., Liang W.M., Jiang R.S., Wang W.Y. (2003). Phase III study of concurrent chemoradiotherapy versus radiotherapy alone for advanced nasopharyngeal carcinoma: Positive effect on overall and progression-free survival. J. Clin. Oncol..

[4] Chua M.L., Wee J.T., Hui E.P., Chan A.T. (2016). Nasopharyngeal carcinoma. Lancet.

[5] Kamran S.C., Riaz N., Lee N. (2015). Nasopharyngeal carcinoma. Surg. Oncol. Clin. N. Am..

[6] Benz M.R., Vargas H.A., Sala E. (2016). Functional MR Imaging Techniques in Oncology in the Era of Personalized Medicine. Magn. Reson. Imaging Clin. N. Am..

[7] Bogowicz M., Vuong D., Huellner M.W., Pavic M., Andratschke N., Gabrys H.S., Guckenberger M., Tanadini-Lang S. (2019). CT radiomics and PET radiomics: Ready for clinical implementation?. Q J. Nucl. Med. Mol. Imaging.

[8] King A.D., Bhatia K.S. (2010). Magnetic resonance imaging staging of nasopharyngeal carcinoma in the head and neck. World J. Radiol.

[9] Zhang Y., Liu X., Zhang Y., Li W.F., Chen L., Mao Y.P., Shen J.X., Zhang F., Peng H., Liu Q. (2015). Prognostic value of the primary lesion apparent diffusion coefficient (ADC) in nasopharyngeal carcinoma: A retrospective study of 541 cases. Sci. Rep..

[10] Zheng D., Yue Q., Ren W., Liu M., Zhang X., Lin H., Lai G., Chen W., Chan Q., Chen Y. (2017). Early responses assessment of neoadjuvant chemotherapy in nasopharyngeal carcinoma by serial dynamic contrast-enhanced MR imaging. Magn. Reson. Imaging.

[11] Xie P., Yue J.B., Zhao H.X., Sun X.D., Kong L., Fu Z., Yu J.M. (2010). Prognostic value of 18F-FDG PET-CT metabolic index for nasopharyngeal carcinoma. J. Cancer Res. Clin. Oncol..

[12] Chen Y.H., Chang K.P., Chu S.C., Yen T.C., Wang L.Y., Chang J.T., Hsu C.L., Ng S.H., Liu S.H., Chan S.C. (2019). Value of early evaluation of treatment response using 18F-FDG PET/CT parameters and the Epstein-Barr virus DNA load for prediction of outcome in patients with primary nasopha-ryngeal carcinoma. Eur. J. Nucl. Med. Mol. Imaging.

[13] Tofts P.S., Berkowitz B., Schnall M.D. (1995). Quantitative analysis of dynamic Gd-DTPA enhancement in breast tumors using a permeability model. Magn. Reson. Med..

[14] Wang H.M., Chen P.T., Chang T.C., Hsu C.L., Lin C.Y., Liao C.T., Chen H. Concurrent chemoradiotherapy using biweekly cisplatin/tegafur plus uracil/leucovorin in stage III nasopharyngeal carcinoma. Proceedings of the Abstracts of the 41st Annual Meeting of the American Society of Clinical Oncology (ASCO).

[15] Kang L., Chen W., Petrick N.A., Gallas B.D. (2015). Comparing two correlated C indices with right-censored survival outcome: A one-shot nonparametric approach. Stat. Med..

[16] Walker-Samuel S., Leach M.O., Collins D.J. (2006). Evaluation of response to treatment using DCE-MRI: The relationship between initial area under the gadolinium curve (IAUGC) and quantitative pharmacokinetic analysis. Phys. Med. Biol..

[17] Lee D.H., Kim S.H., Im S.A., Oh D.Y., Kim T.Y., Han J.K. (2016). Multiparametric fully-integrated 18-FDG PET/MRI of advanced gastric cancer for prediction of chemotherapy response: A preliminary study. Eur. Radiol..

[18] Huang Y.S., Chen J.L., Chen J.Y., Lee Y.F., Huang J.Y., Kuo S.H., Yen R.F., Chang Y.C. (2019). Predicting tumor responses and patient survival in chemoradiotherapy-treated patients with non-small-cell lung cancer using dynamic contrast-enhanced integrated magnetic resonance-positron-emission tomography. Strahlenther. Onkol..

[19] Jain R.K. (1987). Transport of molecules in the tumor interstitium: A review. Cancer Res..

[20] Koh T.S., Ng Q.S., Thng C.H., Kwek J.W., Kozarski R., Goh V. (2013). Primary colorectal cancer: Use of kinetic modeling of dynamic contrast-enhanced CT data to predict clinical outcome. Radiology.

[21] Chin S.C., Lin C.Y., Huang B.S., Tsang N.M., Fan K.H., Ku Y.K., Hsu C.L., Chan S.C., Huang S.F., Li C.H. (2016). Pretreatment Dynamic Contrast-Enhanced MRI Improves Prediction of Early Distant Metastases in Patients With Nasopharyngeal Carcinoma. Medicine.

[22] Chawla S., Kim S., Loevner L.A., Hwang W.T., Weinstein G., Chalian A., Quon H., Poptani H. (2011). Prediction of disease-free survival in patients with squamous cell carcinomas of the head and neck using dynamic contrast-enhanced MR imaging. AJNR Am. J. Neuroradiol..

[23] Ng S.H., Liao C.T., Lin C.Y., Chan S.C., Lin Y.C., Yen T.C., Chang J.T., Ko S.F., Fan K.H., Wang H.M. (2016). Dynamic contrast-enhanced MRI, diffusion-weighted MRI and 18F-FDG PET/CT for the prediction of survival in oropharyngeal or hypopharyngeal squamous cell carcinoma treated with chemoradiation. Eur. Radiol.

[24] Yan D.F., Zhang W.B., Ke S.B., Zhao F., Yan S.X., Wang Q.D., Teng L.S. (2017). The prognostic value of pretreatment tumor apparent diffusion coefficient values in nasopharyngeal carcinoma. BMC Cancer.

[25] Chan S.C., Chang J.T., Lin C.Y., Ng S.H., Wang H.M., Liao C.T., Chang C.J., Lin S.Y., Yen T.C. (2011). Clinical utility of 18F-FDG PET parameters in patients with advanced nasopharyngeal carcinoma: Predictive role for different survival endpoints and impact on prognostic stratification. Nucl. Med. Commun..

[26] Li Q., Zhang J., Cheng W., Zhu C., Chen L., Xia F., Wang M., Yang F., Ma X. (2017). Prognostic value of maximum standard uptake value, metabolic tumor volume, and total lesion glycolysis of positron emission tomography/computed tomography in patients with nasopharyngeal carcinoma: A systematic review and meta-analysis. Medicine.

[27] Chang K.P., Tsang N.M., Liao C.T., Hsu C.L., Chung M.J., Lo C.W., Chan S.C., Ng S.H., Wang H.M., Yen T.C. (2012). Prognostic Significance of 18F-FDG PET Parameters and Plasma Epstein-Barr Virus DNA Load in Patients with Nasopharyngeal Carcinoma. J. Nucl. Med..

[28] Yu H., Mohan S., Natarajan M. (2012). Radiation-Triggered NF-kappaB Activation is Responsible for the Angiogenic Signaling Pathway and Neovascularization for Breast Cancer Cell Proliferation and Growth. Breast Cancer.

[29] Lee N.Y., Zhang Q., Pfister D.G., Kim J., Garden A.S., Mechalakos J., Hu K., Le Q.T., Colevas A.D., Glisson B.S. (2012). Addition of bevacizumab to standard chemoradiation for locoregionally advanced nasopharyngeal carcinoma (RTOG 0615): A phase 2 multi-institutional trial. Lancet Oncol..

[30] Tsao S.W., Tsang C.M., Lo K.W. (2017). Epstein-Barr virus infection and nasopharyngeal carcinoma. Philos. Trans. R. Soc. Lond. B Biol. Sci..

[31] Chan S.C., Ng S.H., Yeh C.H., Chang K.P. (2020). Multiparametric PET/MRI in nasopharyngeal carcinoma: Correlations between MRI functional parameters and 18F-FDG PET imaging biomarkers and their predictive value for treatment failure. Tzu. Chi. Med. J..

[32] Lee V.H., Kwong D.L., Leung T.W., Choi C.W., Lam K.O., Sze C.K., Ho P., Chan W.L., Wong L.S., Leun D. (2016). Post-radiation plasma Epstein-Barr virus DNA and local clinical remission after radical intensity-modulated radiation therapy for nasopharyngeal carcinoma. Clin. Oncol. Coll. Radiol..

[33] Ferrari D., Codecà C., Bertuzzi C., Broggio B., Crepaldi F., Luciani A., Floriani I., Ansarin M., Chiesa F., Alterio D. (2012). Role of plasma EBV DNA levels in predicting recurrence of nasopharyngeal carcinoma in a Western population. BMC Cancer.

[34] Argirion I., Zarins K.R., Ruterbusch J.J., Vatanasapt P., Sriplung H., Seymour E.K., Rozek L.S. (2020). Increasing incidence of Epstein-Barr virus-related nasopharyngeal carcinoma in the United States. Cancer.

